# The effects of progesterone on the healing of obstetric anal sphincter damage in female rats

**DOI:** 10.1515/med-2023-0786

**Published:** 2023-09-01

**Authors:** İrem Şenyuva, Duygu Baki Acar, Hasan Hüseyin Demirel, Ece Tunç

**Affiliations:** Medical Faculty, Department of Obstetrics and Gynecology, Uşak University, Uşak, Turkey; Veterinary Faculty, Department of Obstetrics and Gynecology, Afyon Kocatepe University, Afyon, Turkey; Veterinary Faculty, Department of Pathology, Bayat Vocational School, Afyon Kocatepe University, Afyon, Turkey

**Keywords:** anal canal, injuries, obstetrics, progesterone

## Abstract

We aimed to evaluate the effects of postpartum progesterone on obstetric anal sphincter injury (OASI) healing in female rats using an experimental OASI model. Twenty-eight female rats were divided into four groups after birth: sham-30, sham-90, progesterone (P4)-30, and P4-90. Moreover, OASI model was established in all groups. Subsequently, except for the sham groups, medroxyprogesterone acetate (0.15 mg) was intramuscularly injected into the P4 groups. After 30 and 90 days, the rats were euthanized under general anesthesia after recording the data. The anal sphincter region was collected for histopathological examination. Progesterone and thiol/disulfide homeostasis studies were performed on blood samples. No significant differences were observed between the groups regarding the external anal sphincter (EAS), internal anal sphincter (IAS), or connective tissue thickness (*p* = 0.714, *p* = 0.135, and *p* = 0.314, respectively). No statistically significant differences in the total thiol, native thiol, disulfide, and progesterone levels were found between the groups (*p* = 0.917, *p* = 0.503, *p* = 0.361, and *p* = 0.294, respectively). The endometrial thickness was lower in the P4 groups than in the sham groups (*p* = 0.031). Postpartum progesterone administration did not affect IAS and EAS or connective tissue thickness or disrupt the thiol–disulfide balance. However, this administration led to endometrial thinning.

## 1 Introduction

Anal incontinence (AI) is one of the symptoms of pelvic floor dysfunction that manifests with loss of control of solid and/or fluid and/or gas contents and could negatively impact women’s social and sexual life and prestige [1]. The most common cause of AI in healthy women is obstetric anal sphincter injury (OASI), which has been found to occur in 0.5–9% of all deliveries [1,2].

OASI has been found to affect planning for next pregnancy in women [3]. Subsequent pregnancies have two major risks: recurrent OASI and developing AI. The rate of these risks was found to be 17–24% in the literature [4,5]. Women with OASI had their next pregnancy within 1–2 years: 41% of them did not use contraceptive methods [6]. Pelvic floor recovery after vaginal delivery was observed in the first few months. Thus, the use of contraceptive methods is significant during this period [6,7]. Currently, there are no recommendations for family planning in postpartum women with OASI [6].

Hormonal contraception is a treatment option in postpartum women [8]. However, women with OASI should be evaluated not only for lactation and thrombosis, but also for anal sphincter healing [9]. This is because anal sphincter muscles and their connective tissue contain sex hormone receptors [10]. At this point, only progesterone methods (oral/depo form) are considered safe according to the Medical Eligibility Criteria compared with combined hormonal contraception after the first months of delivery [8]. Progesterone is a hormone exerting a variety of effects on different tissues. Progesterone provides contraceptive effects via suppressing ovulation and endometrial atrophy in the reproductive tract. Moreover, it can increase muscle protein synthesis and mass in the skeletal muscle, enhance antioxidant and anti-inflammatory activities in cells via nuclear factor kappa B (NF-κB) activation, and decrease smooth muscle proliferation [11–14]. However, there is currently no research that examines how postpartum progesterone affects sphincter healing in women with OASI.

Thus, this is the first study to evaluate the effects of progesterone on OASI healing in female rats. The null hypothesis was that there was no relationship between progesterone use, healing of anal sphincter damage, and possible endometrial effects.

## 2 Materials and methods

The animal experiments were performed between January and March 2021 at the Experimental Animal Application and Research Center of Ayon Kocatepe University in Afyonkarahisar, Turkey. The National Guidelines for the Use and Care of Laboratory Animals were followed in the research. The Animal Experiments Local Ethics Committee of Afyon Kocatepe University (Afyonkarahisar, Turkey; decision number 4953702/214 dated 24.02.2020) accepted the study.

### 2.1 Animals and study design

Twenty-eight healthy Sprague-Dawley female virgin rats, 8–12 weeks old (230–280 g), were used. The rats were housed in animal shelters and fed with standard rat chow and tap water in a day/night period of 12 h cycles at a temperature of 21–24°C. After 1-week adaptation period, all rats were mated, and pregnancy and birth processes followed.

### 2.2 OASI model and progesterone administration

**Figure 1:**
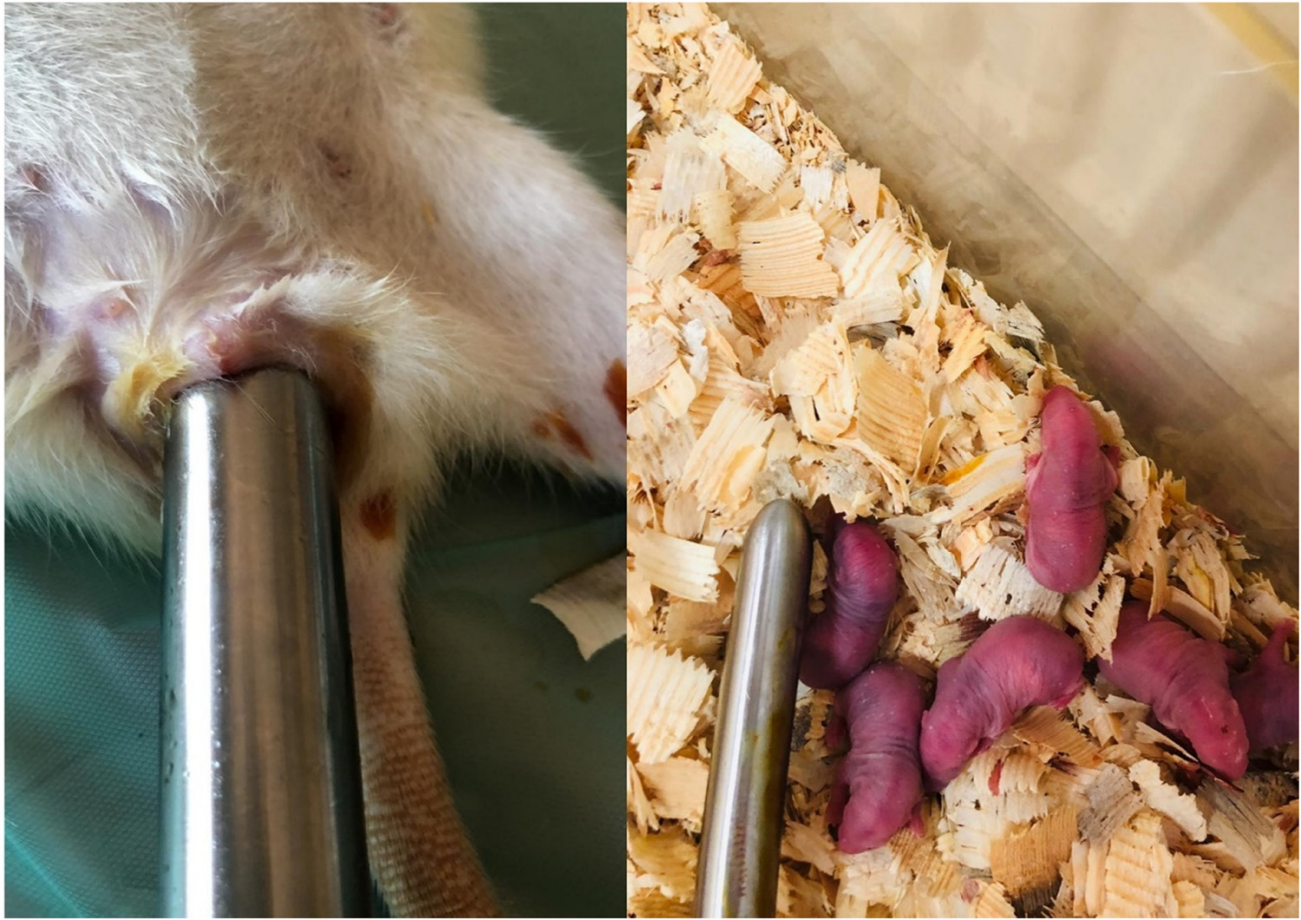
OASI model.

After birth, the rats were divided into four groups: sham-30, sham-90, progesterone (P4)-30, and P4-90. The rats in the OASI model were anesthetized with xylazine (10 mg/kg; Ege Vet, İzmir, Turkey) and ketamine (50 mg/kg; Ege Vet, İzmir, Turkey) administered intraperitoneally. According to this model, a 15 mm cervical dilatator was placed into the vagina for 1 h and then it was removed. Thereafter, anal sphincter damage was achieved with full-thickness internal and external sphincterotomy (Figure 1). The rectal mucosa was sutured with 5-0 braided polyglactin in a double layer of the primary suture. Two sutures, set 1 mm apart, were placed on the anal sphincter muscle layer with 5-0 braided polyglactin [15,16].

Within the scope of a placebo administration, the sham-30 and sham-90 groups received a volume of intramuscular (i.m.) saline injection into the caudal thigh muscle. The rats in the P4-30 and P4-90 groups were injected, i.m., with 0.15 mg of medroxyprogesterone acetate (Depo Provera; Eczacıbaşı Ltd Şti., İstanbul, Turkey) for once on the day of the OASI model [17].

### 2.3 Termination of the experimental model

**Figure 2:**
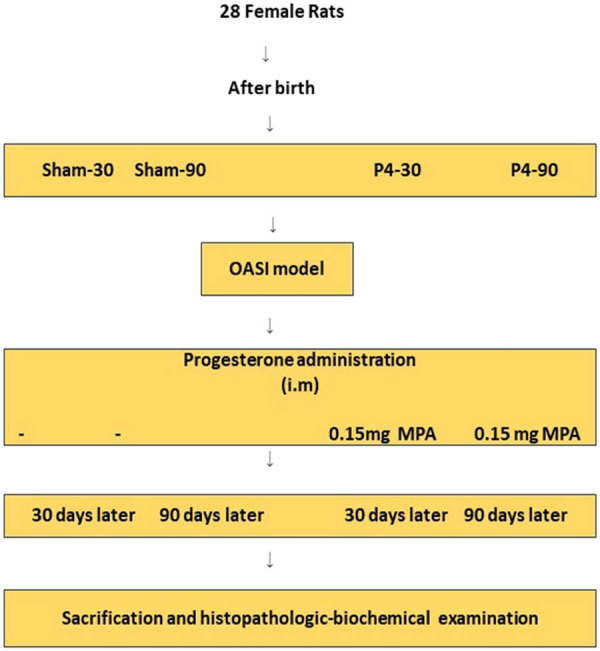
Flow chart of the study. P4: progesterone; OASI: obstetric anal sphincter injury model; MPA: medroxyprogesterone acetate; i.m.: intramuscular.

At the end of the experiment (30 days after plasebo-P4 and 90 days after plasebo-P4), the rats were placed under general anesthesia with xylazine (10 mg/kg; Ege Vet, İzmir, Turkey) and ketamine (50 mg/kg; Ege Vet, İzmir, Turkey) administered intraperitoneally and euthanized by collecting blood from the heart after recording the data. The anal sphincter and uterine tissue samples were preserved in a 10% buffered formaldehyde solution. A histological examination was carried out. Blood samples from the heart were centrifuged at 3,000 rpm for 10 min to separate the sera. Progesterone and thiol/disulfide homeostasis were frozen at −20°C until the day of biochemical examination, and then thawed at room temperature. The OASI experimental model flow chart is shown in Figure 2.

### 2.4 Histopathological examination

**Figure 3:**
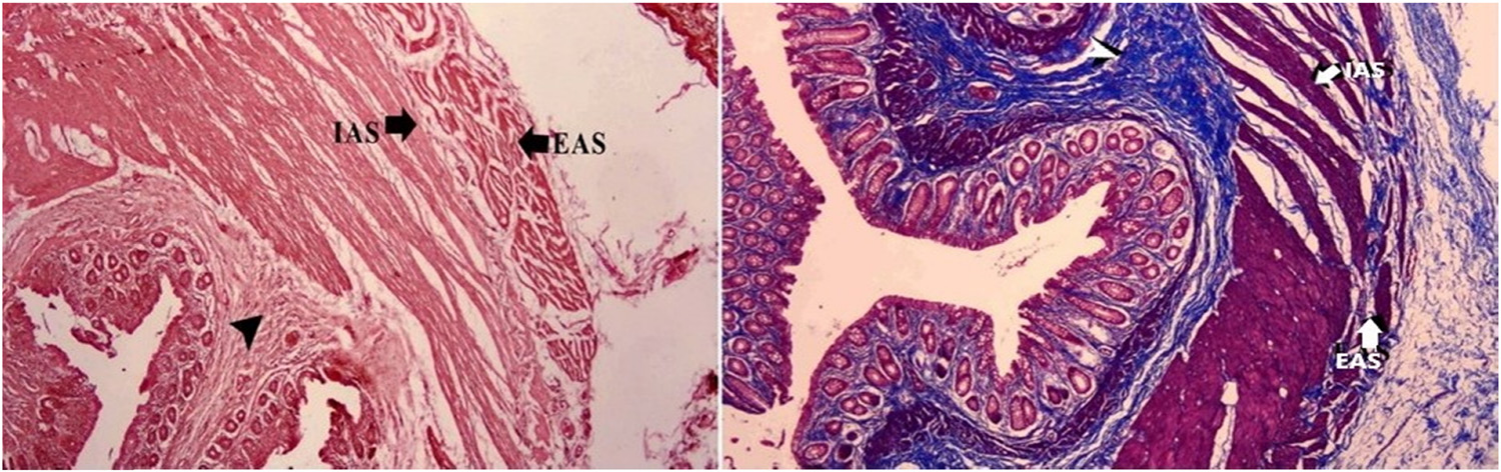
Histopathological section of anal sphincter. IAS: internal anal sphincter; EAS: external anal sphincter; arrow head: connective tissue (H&E: hematoxylin & eosin and Masson trichrome).

Tissue samples after formalin fixation were reduced to 2–3 mm thickness and appropriate sizes and placed into labeled tissue cassettes. After being washed under running tap water overnight, they were maintained in 50, 70, 80, 96% ethanol and xylol, paraffin with xylol, paraffin melted at 56–58°C for 2 h for each procedure, and embedded in paraffin. The samples were cut with a microtome (RM 2245; Leica Biosystems, Deer Park, IL, USA) in 5 µm thickness from each paraffin block were taken to slides by means of a water bath (HI 1210; Leica Biosystems, Germany). They were dried in an oven for 10 min (Thermo Fisher Scientific, Waltham, MA, USA) and prepared for histopathological analysis. All sections were passed through absolute, 96, 80, 70, and 50% ethanol series and xylol series and stained with hematoxylin–eosin and Masson’s trichrome staining method [18]. Stained preparations were examined under a binocular headlight microscope (Eclipse Ci; Nikon, Tokyo, Japan). The transverse widths of the external anal sphincter (EAS), internal anal sphincter (IAS), connective tissue, and thickness of endometrium structures were measured with a photomicrometer (µm) at 10× magnification from four areas where the muscle fibers and connective tissue of the anal sphincter complex were regular and the obtained values were averaged (Nikon DS FI3; microscopic digital camera systems, NIS-Elements, Tokyo, Japan) (Figure 3).

### 2.5 Biochemical analysis

A unique automatic and spectrophotometric approach was used to quantify thiol/disulfide homeostasis (mol/L) [19], and progesterone (pg/mL) levels were measured using a competitive inhibition enzyme immunoassay technique (Rel Assay Diagnostics, Şehitkamil/Gaziantep, Turkey) [20]. Serum samples were thawed at room temperature immediately before biochemical analysis.

### 2.6 Statistical analysis

The Number Cruncher Statistical System 2007 program (Kaysville, UT, USA) was used for statistical analyses. During the evaluation of the study data, descriptive statistical methods (mean, standard deviation, median, frequency, ratio, minimum, and maximum) were used. Data distribution was also evaluated using the Shapiro–Wilk Test. The Kruskal–Wallis test was used for comparing the quantitative data among three or more groups that did not show normal distribution. The Mann–Whitney *U* test was used for comparing between two groups that did not show normal distribution. Significance was set at *p* < 0.01 and *p* < 0.05 levels.

## 3 Results

### 3.1 Anal sphincter muscles and connective tissue thickness

EAS, IAS, and connective tissue thickness did not significantly differ between the groups (*p* = 0.714, *p* = 0.135, and *p* = 0.314, respectively). The results are presented in Table 1.

**Table 1:** IAS, EAS and connective tissue thickness values of the groups

Groups	Tissue thickness (µm)	*p*
**IAS**		0.135
Sham-30	220.61–570.28 (386.46 ± 135.03)	
Sham-90	305.28–596.71 (402.97 ± 100)	
P4-30	165.82–570.57 (335.14 ± 118.7)	
P4-90	169.19–393.48 (280.06 ± 77.28)	
**EAS**		0.714
Sham-30	172.2–411.62 (270.57 ± 88.47)	
Sham-90	147.6–447.99 (269.46 ± 120.18)	
P4-30	143.34–489 (312.38 ± 110.17)	
P4-90	180.91–368.71 (268.73 ± 70.69)	
**Connective tissue**		0.314
Sham-30	302.05–405.86 (359.02 ± 46.26)	
Sham-90	253.51–411.21 (337.19 ± 57.23)	
P4-30	174.1–435.93 (333.07 ± 86.41)	
P4-90	164.58–408.93 (293.4 ± 79.28)	

EAS: external anal sphincter; IAS: internal anal sphincter; P4: progesterone.

### 3.2 Progesterone levels and oxidative stress

Total thiol, native thiol, disulfide, and progesterone levels were not statistically different amongst the groups (*p* = 0.917, *p* = 0.503, *p* = 0.361, and *p* = 0.294, respectively) (Table 2).

**Table 2:** Biochemical values of groups

Groups	Biochemical values	*p*
**Progesterone (pg/mL)**		0.294
Sham-30	12.81–19.52 (16.97 ± 2.37)	
Sham-90	13.02–18.98 (16.44 ± 2.08)	
P4-30	11.19–18.28 (15.08 ± 2.38)	
P4-90	13.24–18.06 (16.54 ± 1.88)	
**TTL (µmol/L)**		0.917
Sham-30	226–801 (687.86 ± 204.56)	
Sham-90	734–798 (764.29 ± 20.65)	
P4-30	736–865 (779.6 ± 50.39)	
P4-90	214–857 (728.8 ± 185.6)	
**NTL (µmol/L)**		0.503
Sham-30	114–294 (196.43 ± 54.63)	
Sham-90	108–237 (174.71 ± 52.84)	
P4-30	38–320 (167.7 ± 70.03)	
P4-90	98–206 (159.3 ± 43.14)	
**Disulfide**		0.361
Sham-30	27–321 (245.71 ± 100.14)	
Sham-90	267.5–325.5 (294.79 ± 22.41)	
P4-30	272–413.5 (305.95 ± 42.12)	
P4-90	38.5–373.5 (284.75 ± 90.77)	

TTL: total thiol level; NTL: native thiol level; P4: progesterone.

### 3.3 Endometrial effect

Table 3 presents the effects on the endometrium. Endometrial thickness measurements were significantly different between the groups (*p* = 0.031). Endometrial thickness in the sham-30 group was higher than that in the P4-30 group (*p* = 0.001), and increased endometrial thickness was observed in the sham-90 group compared with the P4-90 group (*p* = 0.001). The endometrial thickness significantly increased in the P4-30 compared with the P4-90 (*p* = 0.001) group.

**Table 3:** Endometrial thickness of groups

Groups	Endometrial thickness (µm)	*p*
Sham-30	250.76–436.6 (373.43 ± 62.09)	0.031
Sham-90	279.04–462.57 (368.9 ± 59.31)	
P4-30	265.03–477.82 (365.18 ± 71.2)	
P4-90	200.91–358.21 (293.06 ± 52.1)	

P4: progesterone.

## 4 Discussion

Our study demonstrated that progesterone use after delivery did not affect the IAS and EAS muscles and connective tissue thickness or disrupt the thiol–disulfide balance, which is a marker for oxidative stress. However, this use led to endometrial thinning.

The anal sphincter complex consists of the EAS and IAS muscles and their connective tissues [21]. Although IAS smooth muscle and connective tissue contain progesterone receptors (PRs), which provide anal resting pressure, EAS-striated muscle does not contain PRs, which are responsible for voluntary continence [10,21]. Thus, progesterone administration may affect the anal sphincter complex. Progesterone, a sophisticated hormone, exhibits different tissue and treatment regimens and muscle type exhibits different patterns [22–24]. The arterial smooth muscle cells could be inhibited by progesterone (500 nµ) via decreased cell-cycle dependent mechanism. Additionally, the human umbilical vein smooth muscle cells were inhibited by progesterone in physiologic concentrations via mitogen-activated-protein-kinase activity. Although, the aortic vascular smooth muscle cells were increased by progesterone (10–100 nM), longer time decreased this effect [22,25,26]. In contrast, progesterone regulated striated muscle protein synthesis and mass via myogenin and MyoD [12]. Furthermore, high dose (100 mg/day) and long term (1 year) for progesterone treatment increased striated muscle strength and mass [23,24]. Given that the primary null hypothesis could be partially rejected, in our study, we did not observe any effect of progesterone use because PRs were absent in the EAS. However, muscle healing might be related to local paracrine factors [27]. However, we did not detect decreased IAS thickness, which may be explained by the positive effect of progesterone on this tissue. However, the exact mechanism should be further elucidated.

Progesterone exerts an antiproliferative effect on the endometrium by inhibiting epithelial growth [11]. In the literature, following depomedroxyprogesterone acetate administration (150 mg), injectable every 3 months, atrophic endometrium was demonstrated after 3–6 months in 57% [28]. The primary null hypothesis was rejected; in our study, endometrial thinning could be explained by an inhibitory effect.

The pathophysiology of fecal incontinence may be influenced by the loss of myoarchitecture and the replacement of the anal sphincter muscle with fibrotic tissue [29,30]. Research has shown that reactive oxygen species, lipid peroxidation could lead to tissue fibrosis [31]. Progesterone was found to enhance antioxidative enzyme activity and reduce lipid peroxidation and inflammatory cytokine levels [13]. However, the fibrotic and anti-fibrotic effects of progesterone differ among tissues. In the lung, progesterone increases fibrosis via tumor necrosis factor-beta. In cardiac tissues, PR membrane component 1 enhances mitochondrial respiration and protects against cardiac failure. *In vitro* studies (organoids from non-inflamed colonic biopsies), progesterone alleviated wound healing and fibrosis via decreasing interleukin 6 and interleukin 8 levels [32–34]. The primary null hypothesis could be rejected because in our study, progesterone did not cause anal sphincter fibrosis or impair thiol–disulfide levels. This may be explained by the antioxidative and anti-inflammatory effects of progesterone. However, the main molecular mechanism remains to be determined.

The women’s sexual activity generally starts 6 weeks or ovulation occurrence at 4 weeks postpartum in non-breastfeeding women [9]. Moreover, unintended pregnancy could occur in 23% of women not using any contraceptive method postpartum at the first 3 months. However, using contraceptive methods, this rate decreased down to 0.5% [6]. Considering the safety and positive impact of anal sphincter healing, progesterone might be an effective contraceptive option in women with OASI early after delivery. However, *in vivo*, and human studies have shown that functional pelvic floor recovery was completed after vaginal delivery within approximately 6 months [27,35]. According to our study, progesterone use in the first 3 months did not result in anal sphincter disruption. However, further research is needed to determine the long-term effects of postpartum progesterone use.

### 4.1 Study limitation

The physiological changes during pregnancy and their effects on anal sphincter damage were not evaluated.

## 5 Conclusion

The current literature provides information on the effect of progesterone on skeletal and smooth muscle fibers. Furthermore, its effect on oxidative stress in various tissues outside the anal sphincter region has been shown. However, we suggest that our study on anal sphincter healing has revealed for the first time that progesterone did not affect the thickness of the anal sphincter muscle and connective tissue or disturb the thiol/disulfide balance. An endometrial effect was also observed. Progesterone may be an effective contraceptive option in women with OASI early after delivery. To understand the long-term role of progesterone, physiological studies are needed to determine the functional state of the anal canal.
